# GenInfoGuard—A Robust and Distortion-Free Watermarking Technique for Genetic Data

**DOI:** 10.1371/journal.pone.0117717

**Published:** 2015-02-17

**Authors:** Saman Iftikhar, Sharifullah Khan, Zahid Anwar, Muhammad Kamran

**Affiliations:** 1 Department of Computing, School of Electrical Engineering and Computer Sciences, National University of Sciences and Technology, Islamabad, Pakistan; 2 Department of Computer Science, COMSATS Institute of Information Technology, Wah Cantt, Pakistan; University of Catania, ITALY

## Abstract

Genetic data, in digital format, is used in different biological phenomena such as DNA translation, mRNA transcription and protein synthesis. The accuracy of these biological phenomena depend on genetic codes and all subsequent processes. To computerize the biological procedures, different domain experts are provided with the authorized access of the genetic codes; as a consequence, the ownership protection of such data is inevitable. For this purpose, watermarks serve as the proof of ownership of data. While protecting data, embedded hidden messages (watermarks) influence the genetic data; therefore, the accurate execution of the relevant processes and the overall result becomes questionable. Most of the DNA based watermarking techniques modify the genetic data and are therefore vulnerable to information loss. Distortion-free techniques make sure that no modifications occur during watermarking; however, they are fragile to malicious attacks and therefore cannot be used for ownership protection (particularly, in presence of a threat model). Therefore, there is a need for a technique that must be robust and should also prevent unwanted modifications. In this spirit, a watermarking technique with aforementioned characteristics has been proposed in this paper. The proposed technique makes sure that: (i) the ownership rights are protected by means of a robust watermark; and (ii) the integrity of genetic data is preserved. The proposed technique—GenInfoGuard—ensures its robustness through the “watermark encoding” in permuted values, and exhibits high decoding accuracy against various malicious attacks.

## Introduction

Genetic data incorporates sensitive information of organisms and is used for biological processes; for instance, translation, transcription and protein synthesis from DNA to protein. The accurate execution of these biological phenomena depends on the accuracy of genetic codes (input data), intermediate processes and methods involved. For this purpose, input data is handed-over to experts of various relevant domains that involves sharing of digital data. In this context, data may face issues related to ownership rights; therefore, data needs to be right protected. In the US, the right protection of databases storing genetic information is highly recommended at the government level [1], [2]. In such systems, modifications cannot be acceptable so that data integrity remains intact. Therefore, DNA, genes and protein datasets, used for analysis in data mining (and other knowledge discovery) applications, are valuable assets and intolerable to distortions; thus, demand the distortion-free watermarking. Consequently, ownership and integrity (the terms, data integrity and data quality are used interchangeably in this paper) of data is preserved without distortions.

DNA based watermarks are embedded in genetic information synthetically, for the purpose of protection of digital rights (or ownership rights). Research prevailing in this area targets redundant codes such as amino acids and least significant base in DNA-Crypt [3] and least significant bits in image steganography [4] to hide watermark. However, owing to the occurrence of mutations in the organism’s DNA sequence over the time, the watermark stored is lost [5]. DNA-Crypt attempts to correct watermark, in which errors can occur due to mutations in the DNA, with some mutation correction codes such as the Hamming-code or the WDH-code. However, this technique is still unable to correct watermark errors that occur due to mutations in the DNA [6]. Moreover, these mutations can also affect the outcome of biological processes because they usually involve data modifications.

In literature, zero-watermarking techniques (that do not bring any change in the original data) such as [7], [8], [9] and [10] are fragile and distortion-free. Distortion-free watermarking makes sure that the original data does not change its appearance and semantics. The major purpose served by these techniques is to ensure data authentication (and data integrity) by a distortion-free technique. The fragility of these techniques makes them vulnerable to various malicious attacks and hence, they are not appropriate for ownership protection. The main objective of this paper is to present the design of an approach—Genetic Information Guard (GenInfoGaurd), that ensures ownership as well as integrity of genetic data (in this paper, genetic relational databases, genetic data or genetic datasets are same concepts). GenInfoGuard is a robust and distortion-free solution for reversible watermarking of genetic data that encodes a watermark in permuted or hashed values of the original data rather than in the original data values. In this way, the original data remains useful and its integrity is not compromised. The watermark is then verified from watermarked permuted values to claim ownership. Reversible watermarking makes sure that the original data is recovered after watermark decoding. To overcome fragility of distortion-free techniques, the watermark robustness is achieved by permutation matrix calculation, with secret key based encoding (marking) and decoding algorithms. Encoding algorithm has been designed with the consideration of a threat model comprising malicious attacks such as: insertion, deletion, alteration, sorting, additive, and counterfeiting attacks. This threat model was designed with the assumption that an attacker (Mallory) does not have the access to the secret parameters used for marking and secret keys used for digrams (a pair of characters) and permutations computation. For such a watermarking technique, there is no need to store original version of data. The “encoded” watermark is registered as a certificate for verification, with Certification Authority (CA). Mallory would not be able to create the same certificate as her own watermark to claim ownership. Moreover, the permutations performed for watermark encoding are based on usability constraints of data owner and ensure data integrity.

GenInfoGuard mainly comprises a data preprocessing, encoding and decoding phases. In data preprocessing phase, a feature to be marked is selected, a user defined watermark string is generated, secret keys are defined, and the matrices for digrams and permutation of digrams are computed. The permutations are performed with owner defined secret keys. In the encoding phase, genetic codes (non-numeric values) are substituted from the selected feature on the basis of a computed permutation matrix. The substituted values are encoded with a watermark, and the watermarked data for intended recipients is generated. In the decoding phase, preprocessing steps are performed again and decoding strategies are used to recover the watermark without using original data. The major contribution of our work is a robust and distortion-free watermarking technique that ensures ownership protection along with data integrity. GenInfoGuard is resilient to malicious attacks and benign mutations. Therefore, GenInfoGuard ensures that the results of all biological procedures applied to the marked data are accurate. The robustness of GenInfoGuard has been evaluated with experiments based on the attacks defined in the threat model in Section 3.2. The effectiveness and the feasibility of GenInfoGuard has been demonstrated with the real life datasets.

The subsequent sections of the paper are structured as follows. In the Section 2, a brief overview of the state-of-the-art is presented by emphasizing different directions of our work. Section 3 provides the detail about the threat model. In the Section 4, the proposed scheme is described in detail. The Section 5 contains the discussion on experiments and results with the examples of protein, and DNA datasets. Finally, the paper is concluded in the Section 6 with an outlook to future work.

## Related Work

Genetic data is stored in relational databases that are used in biological processes [11], [12]. As the format of the genetic data is usually non-numeric, existing literature on watermarking of non-numeric simple data as well as genetic data was reviewed. Some of the existing techniques on watermarking of non-numeric data were evaluated for providing robust and distortion-free watermarking. Existing watermarking techniques providing ownership protection of non-numeric relational data, modify the data, and result in permanent distortions. In addition, modifications are not secure against attackers. These techniques rely on minor modifications to the actual text characters such as, changing the case of the word(s), adding some white spaces, or ordering the tuples in ascending or descending manner.

DNA based watermarking techniques, usually, hide watermark information in the least significant bits (LSB) and the least significant base of amino acids. However, it results in the watermark loss with the occurrence of mutations in the DNA sequence.

As far as the literature for watermarking of genetic data is concerned, more work has been done on the data of artificial organisms with dummy strands [13], [14] [15], as compared to living organisms’ data [16], [5], [3], [4], [6]. In [13], Clelland et al. proposed a scheme that hides synthetic DNA sequence in English alphabets during encoding. The receiver can extract the DNA sequence from the mappings of key based translations between DNA codons and English alphabets while decoding. In [14], Gehani et al. proposed a cryptographic scheme, based on one-time-pad for synthetic DNA. In [15], Leier et al. designed a steganographic scheme by hiding DNA dummy strand into message sequence and a receiver can extract the original sequence with the help of a key that is another DNA strand. In [16], Arita et al. produced a watermarking method whereby a watermark is embedded in the redundant code or amino acids on the bases of synonyms. In all these techniques, a major drawback is that, if mutations take place in the DNA sequences with the passage of time, the watermark stored in the DNA sequences is lost. In [3], [4], [6], Heider and Barnekow proposed cryptographic techniques which work on the basis of small redundant codes in the data of living DNA strands. The purpose of these techniques is to correct the watermark having errors due to mutations in the DNA. However, the general approach used in these techniques is still unable to provide robust and distortion free watermarking for DNA data.

Other relevant literature include techniques for categorical data. A technique for ownership rights protection of categorical data was provided by Sion et. al, in [17] and [18]. This technique incorporates cryptographic hash function and a secret key for selection of tuples to be watermarked. Another watermarking technique for medical categorical data proposed in [19] is based on histogram shifting, computed from values. Other such techniques include [7], [8], [9] and [10]. Such techniques are not robust against malicious attacks.

Techniques like [20], [21], [22], [23], [24], [25], and [26] bring some distortions in the dataset and therefore they are not suitable for watermarking of genetic data.

To the best of our knowledge, there is not a single technique for genetic data that can provide robust and distortion-free watermarking.

## Threat Model

This section systematically presents the facts regarding the system and adversary capabilities; thus, encompasses all the possible scenarios with the perspective of an attacker.

### 3.1 System Model

Data owner—Alice possesses relational data comprising of genome sequences. Without loss of generality, we assume that the data is non-numeric in the form of strings stored as tuples in a database. She wants to distribute this data to experts (Bob) of various relevant domains and some of them might also aim to steal the data and resell it as their own. She possesses necessary skills, technologies and time to watermark her data before distribution. Watermarking technique is not allowed to modify the data in any way; otherwise, its meaning would be lost and it would become useless. The domain experts, to whom the data is distributed, may run certain experiments on the data which may cause mutations and must not destroy the watermark. She should be able to recover the watermark from the data no matter how much benign mutations and malicious attacks modify it. She should be able to detect what fraction of tuples and DNA strands have been tampered with, when processing tampered data. She should be able to recover watermark and the original data. She does not need to keep a copy of the original data for two reasons: (1) watermark is embedded in the original data by encoding its permuted values; and (2) watermark decoding does not require it for watermark decoding (or recovery). She should input: (1) a secret threshold for feature selection; (2) a seed value for watermark string generation; (3) a secret key of number of characters and symbols for computing digram matrix; and, (4) a secret number of rounds (defined by the owner) for introducing randomness in the permutation matrix. There is no need to store digram and permutation matrix as these can be trivially regenerated while restoring watermark and the original data. She should define an integer value and a percent for watermark encoding and decoding. We assume that all the secret parameters and keys are not compromised and attackers cannot reproduce some or all of them. She will not share these secret parameters and keys with anyone.

### 3.2 Adversary Model

The intention of the adversary, Mallory, is to corrupt (or remove) the watermark from the marked dataset of Alice. It is assumed that in various types of malicious attacks, Mallory tries to destroy watermark ***W*** of Alice. These attacks consist of insertion, deletion, alteration, sorting, additive and counterfeiting attacks, defined in Table 1. Alice has to make her watermark robust against these attacks and she must be able to successfully extract the encoded watermark from the attacked dataset.

**Table 1:** Malicious Attacks.

**Attack**	**Name**	**Description**
*A*_1_	Insertion attacks	Insert *α* new tuples
*A*_2_	Deletion attacks	Delete *α* old tuples
*A*_3_	Alteration attacks	Alter *α* old tuples
*A*_4_	Sorting attacks	Sort the tuples in ascending or descending order
*A*_5_	Additive attacks	Embed own computed watermark in the database, and claims ownership
*A*_6_	Counterfeiting attacks	Make a forged copy of the Alice’s data, for un-authorized use.

Apart from the defined types of attacks, there are some approaches for *A*_2_ and mix-match attacks that should be considered through GenInfoGuard. Approaches for deletion attacks are horizontal sampling and vertical sampling. In horizontal sampling, Mallory deletes some tuples from the watermarked data horizontally, if she gets successful in intuitively identifying data having no watermark embedded and then she sells the remaining tuples illegally. In vertical sampling, Mallory deletes some portion of the data vertically and left with rest of the non-watermarked features, might be useful for someone. So, she sells it illegally. In mix-match attack, she attempts to make her own dataset by combining subsets from different but similar datasets. Her intention is to claim the ownership of the newly created dataset.

The capabilities of Mallory include:

1. She has the access of watermarked dataset but not to the original data ***D*** and watermark ***W***.
2. She has the access of GenInfoGuard to identify watermark encoding and decoding.
3. She is sufficiently motivated to alter the watermark even if it takes long time duration.

## GenInfoGuard Architecture

**Fig 1:**
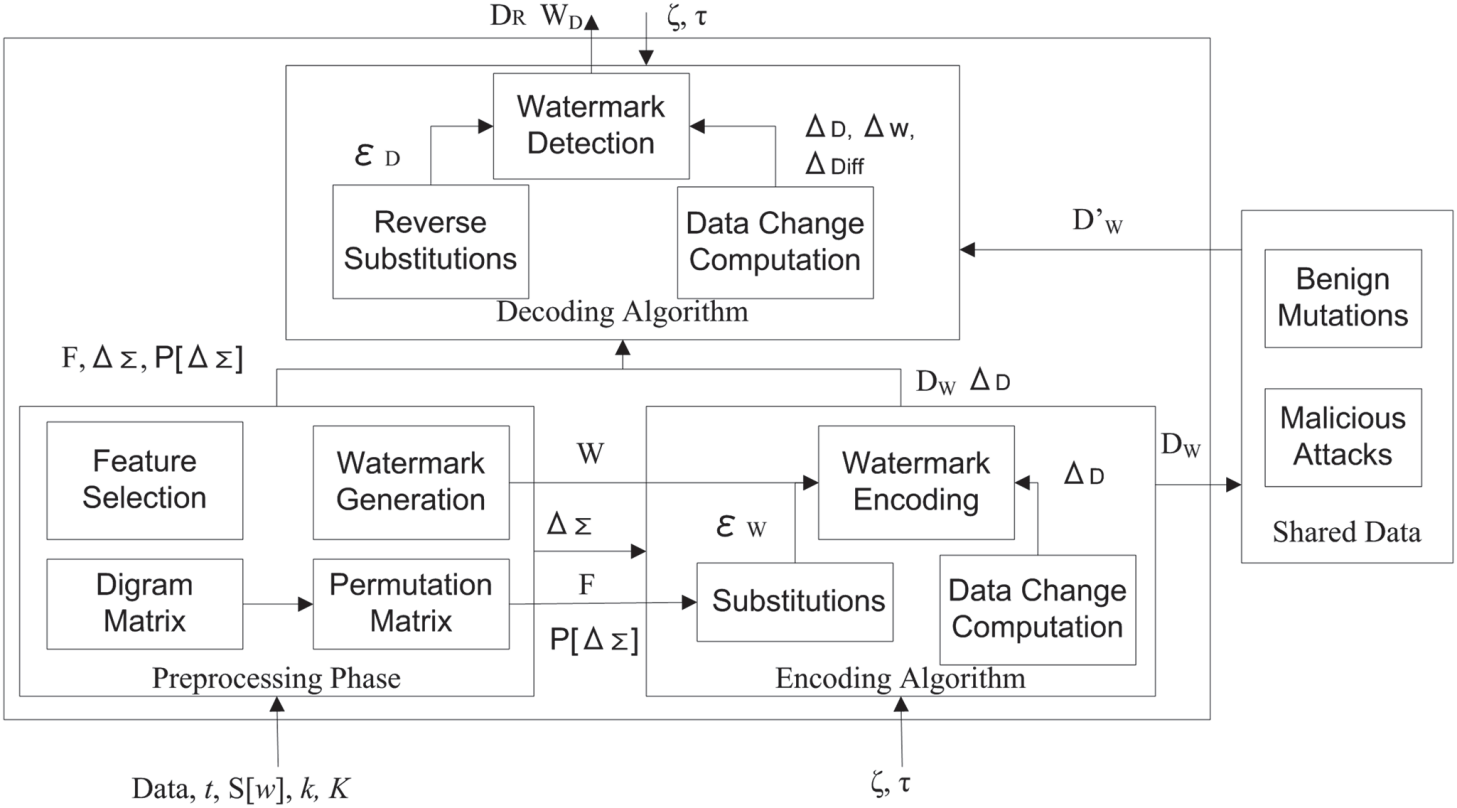
Top Level Architecture of GenInfoGuard.

A top level architecture of GenInfoGuard has been shown in the Fig. 1. It encompasses a preprocessing phase, watermark encoding (marking) and decoding. In the figure, the shared data is a separate place for sharing the watermarked data with the research community and the attacker can also have the open access to this data. Benign mutations are the changes occur in the genetic data over the time while malicious attacks are the changes bring into the data deliberately to destroy the encoded watermark and the original data. Firstly, in the preprocessing phase, we have used mutual information *MI* for feature selection because *MI* can be used to measure the correlation among genes, amino acids, protein structure and genome sequences. If we consider *MI* while encoding a feature with watermark, the statistical information hidden in the genetic data will get preserved. A relatively high impact feature *F* having *MI* more than the secret threshold *t* has been selected for watermarking such that the rest of the data could not remain useful after deleting the watermarked feature. Secondly, watermark string has been generated for encoding. Thirdly, digram matrix and permutation matrix are computed to be used in watermark encoding and decoding. There is no need to store both matrices and can be regenerated when required for encoding and decoding. Permutations are generated randomly with Alice’s defined number of rounds and it make the task of Mallory more difficult. Next, each digram from all tuples of the selected feature has been encoded with the watermark; as a result, it is very difficult for Mallory to destroy the watermark from each digram of each tuple. She cannot destroy the whole data; therefore, she will not succeed in deleting (or removing) the watermark. While encoding watermark in the permuted digrams, a matrix of change data amount is needed to store for verifying watermark at detection side. Finally, change amount computation during watermark encoding and decoding will help in verifying the encoded watermark.

For a quick reference, the Table 2 lists the notations used in this paper.

**Table 2:** Notations Used in the Paper.

**Symbol**	**Description**
*D*	Original database
*D*_*W*_	The watermarked database
*R*	All tuples in a table (or a dataset)
*r*	A tuple in the database table
*t*	Owner defined threshold for *MI* of all features
*m*	All features in dataset
*S*[*ω*]	A Seed vector for Pseudo-Random Sequence Generator
*F*	A feature selected for watermarking
*β*	Determines character at a specified index
*γ*	Index of a specified character in permuted matrix
*k*	A key based on number of characters used in digram matrix
*κ*	Secret number of rounds for permutations defined by the owner
Σ	All accepted characters
Δ_Σ_	Matrix of all possible digrams
*ℓ*	Length of input data string
*ℓ*_Δ_Σ__	Length of the matrix of all possible digrams
*ρ*	Index of the character in digram matrix
*P*[*N*]	Permutation vector for introducing randomness N times
*ω*	Watermark length
*b*	Watermark bits
*ζ*	Owner defined an integer change
*τ*	Owner defined percentage change
Δ_*D*_	Change in the original value
Δ_*D*_*W*__	Change in the watermarked value
Δ_*Diff*_	Difference in change in the original value and the watermarked value
*ξ*_*W*_	Substituted digram for encoding
*ξ*_*D*_	Decoded data from Substituted digram
℘	Probability of success of prediction
℘_*O*_	Probability of success of prediction before applying watermarking
℘_*W*_	Probability of success of prediction after applying watermarking
Ç_*α*(*O*)_	Correlation coefficient of *α* helix prediction before watermarking
Ç_*α*(*W*)_	Correlation coefficient of *α* helix prediction after watermarking
Ç_*β*(*O*)_	Correlation coefficient of *β* strand prediction before watermarking
Ç_*β*(*W*)_	Correlation coefficient of *β* strand prediction after watermarking

In the subsequent sections, preprocessing phase, encoding and decoding algorithms of GenInfoGuard are discussed in detail.

### 4.1 Preprocessing Phase

In the preprocessing phase, four important tasks are accomplished: (1) selection of a suitable feature for marking; (2) computation of a watermark string; (3) computation of a digrams matrix along with digrams indices determination; and (4) computation of a permutation matrix with the improvement in the current polygram substitution techniques. This phase has been explained in the following subsections.

#### 4.1.1 Feature Ranking and Selection

Mutual information *MI* is computed to evaluate the importance of a feature in biological processes [27] for watermarking. In GenInfoGuard, features from the dataset to be watermarked are ranked on the basis of their mutual dependence on other features. *MI* indicates the importance of various features in the information extraction processes. *MI* of every feature with all other features is calculated by using Equation 1.

$$\begin{array}{c} MI\left(A,B\right)=\sum_{a}\sum_{b}P_{AB}\left(a,b\right)log\frac{P_{AB}\left(a,b\right)}{P_{A}\left(a\right)P_{B}\left(b\right)} \end{array}$$ (1)

Where, *MI*(*A*, *B*) measures the degree of correlation of features by measuring the marginal probability distributions as *P*_*A*_(*a*), *P*_*B*_(*b*) and the joint probability distribution *P*_*AB*_(*a*, *b*). Alice defines a secret threshold *t* over the values of *MI* of all the features in a dataset and select a feature for watermarking. The mutual information of each feature is stored as *MI* = {*MI*_1_, *MI*_2_, …, *MI*_*m*_} in ascending order. Owner inputs *t* where *MI*_1_ < *t* < *MI*_*m*_. A relatively high impact feature *F* having *MI* more than the secret threshold *t* is selected to be watermarked.

#### 4.1.2 Computation of a Watermark String

For the purpose of embedding a watermark string into a feature, a watermark string is created through a pseudo-random sequence generator [28]. A seed vector *S*[*ω*], with watermark of length *ω*, is computed in this manner. For experimentation purpose, the length of the watermark string *ω* is 16 bits of 0’s and 1’s; however, other watermark lengths can also be chosen.

#### 4.1.3 Computation of a Matrix for Digrams

Initially, a matrix Δ_Σ_ is generated with all possible pair (two characters or digrams) combinations. A key *k* based on number of characters from A to Z and a to z, used in digram matrix is defined by Alice and she can also use any one or all special characters for increasing the key space. This matrix is then provided as an input for the computation of a permutation matrix *P*[Δ_Σ_]. A step by step procedure for digrams matrix computation has been presented in Algorithm 1.

**Algorithm 1** Digrams Matrix Computation

**Input:** *k*, Σ

**Output:** Δ_Σ_

 Δ_Σ_ = *k* * *k*

 // k X k matrix is initialized

**for** *i* = 0 to *k* **do**

   //loop will iterate for all characters

**for** *j* = 0 to *k* **do**

    //loop will iterate for all characters

*β*(*i*) = Σ(*i*)

*β*(*j*) = Σ(*j*)

    Δ_Σ_(*i* * *k* + *j*) = *β*(*i*)∥*β*(*j*)

**end for**

**end for**

 return Δ_Σ_

#### 4.1.4 Computation of a Permutation Matrix

In this step, permutations are performed to substitute the original characters with permuted characters [29]. The permutation matrix *P*[Δ_Σ_] is then computed algorithmically by using previously calculated matrix of digrams Δ_Σ_. Thereafter, permutations are performed. A 1 × 10 vector *P*[*N*] is initialized randomly that introduces randomness in the resultant permutation matrix *P*[Δ_Σ_] by manipulating *P*[*j*] and *P*[*j* + 1] values from *P*[*N*] alternatively. The permutations are computed using Equation 2 for a pre-specified number of times according to a secret number—*κ*, specified by Alice. This step is performed to bring randomness in the initial digrams matrix Δ_Σ_.

$$\begin{array}{c} \begin{array}{cc} & \Delta_{\Sigma }\left(\left(i*k+P\left[j\right]*\kappa \right)mod\ell_{\Delta_{\Sigma }}\right)\\ & \Delta_{\Sigma }\left(\left(\left(\ell_{\Delta_{\Sigma }}-i\right)+P\left[j+1\right]*\kappa \right)mod\ell_{\Delta_{\Sigma }}\right) \end{array} \end{array}$$ (2)

Where *i* = 0 to *ℓ*_Δ_Σ__ and *j* = 0 to *P*[*N*].*length*.

A step by step procedure for the calculation of permutation matrix has been presented in Algorithm 2.

**Algorithm 2** Permutation Matrix Calculation

**Input:** *κ*, *P*[*N*], Δ_Σ_

**Output:** *P*[Δ_Σ_]

*P*[*N*] = *P*[1], …, *P*[*N*]

 // permutation vector is initialized to create randomness in resulting matrix

**for** *i* = 0 to *ℓ*_Δ_Σ__ **do**

   //loop will iterate for all digrams

**for** *j* = 0 to *P*[*N*].*length* **do**

    //loop will iterate for *P*[1], …, (*P*[*N*]/2)

*P*[1] = *P*[2], *P*[2] = *P*[3], *P*[2] = *P*[1] // swap digrams

    permutations are computed as in equation 2

**end for**

**end for**

 return *P*[Δ_Σ_]

### 4.2 Encoding Algorithm

In the encoding phase, all the tuples of the selected feature are get marked by using permutation matrix *P*[Δ_Σ_]. Digram is taken from the value of each tuple and substituted with the digram found at the intersection of the row (*char*1) and column (*char*2) in the permutation matrix. The index of each selected digram *γ*(*D*_*i*_) and *γ*(*D*_*i*+1_) from the feature value is computed with the help of the index of each digram *ρ*(*D*_*i*_) and *ρ*(*D*_*i*+1_) from digram matrix using Equation 3.

$$\begin{array}{c} \begin{array}{cc} & \gamma \left(D_{i}\right)=\rho \left(D_{i}\right)\\ & \gamma \left(D_{i+1}\right)=\rho \left(D_{i+1}\right) \end{array} \end{array}$$ (3)

A watermark string of length *ω* = 16 bits is computed using the pseudo-random sequence generator with a seed vector *S*[*ω*] of 0’s and 1’s. Other values for *ω* can also be used with GenInfoGuard such as 3, 5, 8, 16, 32, 64. If the watermark bit is 1, data will be permuted and change in data values will be computed by using Equation 4. If the watermark bit is 0, data will be permuted and change in data values will be computed by using Equation 5. Each digram is encoded with the watermark; consequently, it is very difficult for Mallory to destroy the watermark. Here, Alice’s specified parameters *ζ* and *τ* are used to compute changes in data values Δ_*D*_ by marking the original data. The computed changes in the data values Δ_*D*_ is saved during watermark encoding to use it while decoding watermark. Finally, Δ_*D*_ is a resultant matrix having less size as compared to the original data. One of the benefits of saving such information is that Alice do not need to store the original data. And when the ownership of the data will need to be proved, Alice can use this part of the data for decoding of watermark bits.

$$\begin{array}{c} \begin{array}{c} \xi_{W}=\Delta_{\Sigma }\left(\gamma \left(D_{i}\right)*k+\gamma \left(D_{i+1}\right)+\zeta \right)\\ \Delta_{D}=\left(\gamma \left(D_{i}\right)*k+\gamma \left(D_{i+1}\right)+\zeta \right)*\tau \end{array} \end{array}$$ (4)

$$\begin{array}{c} \begin{array}{c} \xi_{W}=\Delta_{\Sigma }\left(\gamma \left(D_{i}\right)*k+\gamma \left(D_{i+1}\right)-\zeta \right)\\ \Delta_{D}=\left(\gamma \left(D_{i}\right)*k+\gamma \left(D_{i+1}\right)-\zeta \right)*\tau \end{array} \end{array}$$ (5)

After watermark encoding, new positions of digrams are identified by using key *k*, *ρ*, and *β*, based on number of characters used in the digrams matrix using Equation 6.

$$\begin{array}{c} \begin{array}{cc} & \beta (D_{i})=\rho /k\\ & \beta (D_{i+1})=\rho modk-\zeta \end{array} \end{array}$$ (6)

In our permutation approach for encoding, every character in the tuple values is mapped differently; as a result, the permuted data values get encoded with a watermark. Thus, it makes the watermark robust. The steps for marking the data are listed in Algorithm 3.

**Algorithm 3** Encoding Algorithm

**Input:** *D, k, ℓ, ω, b, P*[Δ_Σ_]

**Output:** *D*_*W*_

**for** *r* = 1 to *R* **do**

   //loop will iterate for all tuples of the database

**for** *i* = 0 to *l* **do**

    //loop will iterate for each value of selected feature

    // index of input data is selected for watermarking

    using eq 3

**for** *b* = 1 to *ω* **do**

     Mark the data by using Equation 4 if the watermark bit is 1

     Mark the data by using Equation 5 if the watermark bit is 0

**end for**

**for** *j* = 0 to *ℓ*_Δ_Σ__ **do**

**if** Δ_Σ_(*j*) == *ξ*_*W*_ **then**

*ρ* = *j*

**end if**

**end for**

    // digrams at position i and i+1 are identified

    using equation 6

*D*_*W*_ // watermarked data

    // computed digrams are concatenated

**end for**

**end for**

 return *D*_*W*_

### 4.3 Decoding Algorithm

In the decoding phase, the watermark is decoded (or verified) by using the substitutions from permutation matrix *P*[Δ_Σ_]. The watermarked data is again processed as digrams and these digrams are decoded back to the original data. The positions of the watermarked data *D*_*W*_*i*__ and *D*_*W*_*i*+1__ are selected to decode using calculation using Equation 7

$$\begin{array}{c} \begin{array}{cc} & \beta (D_{W_{i}})=\rho /k\\ & \beta (D_{W_{i+1}})=\rho modk+\zeta \end{array} \end{array}$$ (7)

The index of the watermarked digrams are again computed using Equation 8.

$$\begin{array}{c} \begin{array}{cc} & \gamma \left(D_{W_{i}}\right)=\rho \left(D_{W_{i}}\right)\\ & \gamma \left(D_{W_{i+1}}\right)=\rho \left(D_{W_{i+1}}\right) \end{array} \end{array}$$ (8)

The decoding algorithm uses the same secret parameters *k* and *ℓ*. The original data is recovered with the help of tracing the characters at the index of positions of encoded digrams in the permuted matrix *P*[Δ_Σ_]. For watermark detection, the change in the watermarked data values Δ_*D*_*W*__ is computed using Equation 9. Alice’s specified parameters *ζ* and *τ* are used to compute change in data values Δ_*D*_*W*__ during decoding.

$$\begin{array}{c} \Delta_{D_{W}}=\left(\gamma \left(D_{W(i)}\right)*k+\gamma \left(D_{W(i+1)}\right)+\zeta \right)*\tau \end{array}$$ (9)

Any discrepancy in change in value Δ_*Diff*_ of the original Δ_*D*_ and the watermarked data value Δ_*D*_*W*__ is computed using Equation 10.

$$\begin{array}{c} \Delta_{Diff}=\Delta_{D_{W}}-\Delta_{D} \end{array}$$ (10)

If Δ_*Diff*_ is less than zero, the decoding algorithm will compute permuted values of digrams using Equation 11, and the decoded watermark bit will be 1. Otherwise, it will compute permuted values of digrams using Equation 12, and the watermark bit will be decoded as 0. Watermark bits are extracted in reverse order, that is, the last encoded bit is decoded first because the effect of last embedded bit should be examined first.

$$\begin{array}{c} \xi_{D}=\Delta_{\Sigma }\left(\gamma \left(D_{W(i)}\right)*k+\gamma \left(D_{W(i+1)}\right)-\zeta \right) \end{array}$$ (11)

$$\begin{array}{c} \xi_{D}=\Delta_{\Sigma }\left(\gamma \left(D_{W(i)}\right)*k+\gamma \left(D_{W(i+1)}\right)+\zeta \right) \end{array}$$ (12)

A step by step pseudo-code for decoding the watermark has been presented in Algorithm 4.

**Algorithm 4** Decoding Algorithm

**Input:** *D_W_, ℓ*

**Output:** *D, detected watermark bit* (*dtW*)

**for** *r* = 1 to *R* **do**

   //loop will iterate for all tuples of the database

**for** *i* = 0 to *l* **do**

     //loop will iterate for each watermarked tuple

*D*_*W*_ = *D*_*W*_*i*__∥*D*_*W*_*i*+1__

**for** *j* = 0 to *ℓ*_Δ_Σ__ **do**

**if** Δ_Σ_(*j*).*equals*(*D*_*W*_) **then**

*ρ* = *j*

**end if**

**end for**// end for j

**for** *b* = *ω* to 1 **do**

      // data at position i, i+1 is selected to decode

      using equation 7

      // index of watermarked data is computed for watermark detection

      using equation 8

      Δ_*D*_*W*__ = (*γ*(*D*_*W*(*i*)_) * *k* + *γ*(*D*_*W*(*i*+1)_) + *ζ*) * *τ*

      Δ_*Diff*_ = Δ_*D*_*W*__ − Δ_*D*_

**if** Δ_*Diff*_ ≤ 0 **then**

        detected watermark bit (dtW) is 1

equation 11

**end if**

**if** Δ_*Diff*_ > 0 and Δ_*Diff*_ ≤ 1 **then**

        detected watermark bit (dtW) is 0

equation 12

**end if**

      // watermarked data is recovered

**end for**// end for i

**end for**// end for b

**end for**// end for r

return *D, dtW*

## Results and Discussion

In this section, GenInfoGuard has been evaluated for providing: (1) distortion-free watermark encoding and decoding and (2) robustness against malicious attacks.

For brevity, experiments have been reported with two genetic datasets (protein dataset and DNA dataset) and a relatively small watermark that consist of 8-bits. The protein dataset used for prediction of secondary structure of proteins in molecular biology [30] contains categorical data and has been shown in the Table 3. The DNA dataset titled “splice-junction gene sequence” [31] has been shown in the Table 4. In protein dataset, the first feature *F* (selected on the basis of *MI* for watermarking) determines 3 character codes for the representation of 20 amino acids, the second feature represents categorical values for detection of secondary structure of proteins where E stands for *β*-strand and H for *α*-helix. There are some other numerical features as well in the dataset that are not shown here for brevity.

**Table 3:** Protein Dataset.

***F***	***Secondary Structure***	***Weights***
GLY	E	0.20
TYR	H	0.49
VAL	H	0.17
THR	E	0.12
PRO	H	0.52
MET	H	0.32
ASP	E	0.26
…	…	…

**Table 4:** DNA Dataset: Splice-Junction Gene Sequences.

***Class***	***Record Name***	***F***
EI	ATRINS-DONOR-521	CCAGCTGCATCACAGGAGGCCAGCGAGCAGG
		TCTGTTCCAAGGGCCTTCGAGCCAGTCTG
EI	ATRINS-DONOR-905	AGACCCGCCGGGAGGCGGAGGACCTGCAGGG
		TGAGCCCCACCGCCCCTCCGTGCCCCCGC
…	…	…
IE	ATRINS-ACCEPTOR-701	TTCAGCGGCCTCAGCCTGCCTGTCTCCCAGG
		TCTCTGTCCTTCCACCATGGCCCTGTGGA
IE	ATRINS-ACCEPTOR-1678	GGACCTGCTCTGCGTGGCTCGCCCTGGCAGTGGGG
		CAGGTGGAGCTGGGTGGGGGCTCTA
…	…	…

DNA dataset contains 3190 tuples and 62 features. The first feature is a class determining whether 60 DNA nucleotides are donors (EI) or acceptors (IE), second feature is a record name and 60 other features (termed as one feature) are DNA nucleotides that were selected for watermarking on the basis of *MI*. For brevity, 60 nucleotides have been shown in one column in the table. The effect of GenInfoGuard on various biological processes was tested with CLC bioinformatics database tool 2.0.1 [32]. The results of GenInfoGuard on these datasets are discussed in the following sub-sections.

### 5.1 Distortion-Free, Reversible Watermarking

For brevity, preprocessing phase, watermark encoding, and decoding are demonstrated only for protein dataset, to give an insight of how GenInfoGuard works. The effect of GenInfoGuard on statistical and predictions measures is discussed for both datasets in the following sub-sections.

#### 5.1.1 Preprocessing Phase

In this phase, a feature *F* of protein dataset was selected for marking on the basis of *MI*. A watermark string of 8-bits was generated with seeded pseudo-random sequence generator to encode all the tuples of the selected feature. A digram matrix Δ_Σ_ was computed with all possible pair combinations and permutation matrix *P*[Δ_Σ_] was generated from the digram matrix Δ_Σ_.

#### 5.1.2 Watermark Encoding

In this phase, protein dataset was taken under consideration, with only 3 tuples of the selected feature *F* to show the whole procedure of watermark encoding. The watermark encoding process has been explained through the Table 5. A digram GL from GLY the value of the first tuple was encoded with watermark bits. Watermark bits have been represented with *b* of length 8. For every bit the permuted digrams *ξ*_*W*_ and changes in data values Δ_*D*_ were computed for digram GL. The same procedure was repeated for the rest of the tuples of the selected feature *F*.

**Table 5:** Watermark Encoding.

***F***	***b***	**1**	**0**	**1**	**1**	**1**	**1**	**0**	**0**
GLY	*ξ*_*W*_	F	HO	F	F	F	F	HO	HO
	Δ_*D*_	17.5	17.1	17.5	17.5	17.5	17.5	17.1	17.1
TYR	*ξ*_*W*_	QI	UJ	QI	QI	QI	QI	UJ	UJ
	Δ_*D*_	53.9	53.5	53.9	53.9	53.9	53.9	53.5	53.5
VAL	*ξ*_*W*_	DD	YG	DD	DD	DD	DD	YG	YG
	Δ_*D*_	56.9	56.5	56.9	56.9	56.9	56.9	56.5	56.5

#### 5.1.3 Watermark Decoding

To show the intuition to how the decoding algorithm works, the same 3 tuples of the selected feature *F* were considered. The steps of watermark decoding have been shown in the Table 6. The changes in the original data values Δ_*D*_ computed during the encoding phase were used in the decoding phase. The changes in the encoded data values Δ_*D*_*W*__ were computed while decoding the watermark from the watermarked data. The Difference in change in the original data values and the encoded data values Δ_*Diff*_ was computed using Equation 10. On the basis of these computed differences, watermark bits *b* were extracted in reverse order and permuted digrams *ξ*_*D*_ for digram GL were decoded accordingly. Finally, the original data value GLY was computed from permuted digrams. The same procedure was repeated for the rest of the tuples of the selected feature *F*.

**Table 6:** Watermark Decoding.

***F***									
GLY	Δ_*D*_	17.1	17.1	17.5	17.5	17.5	17.5	17.1	17.5
	Δ_*D*_*W*__	17.5	17.5	17.5	17.5	17.5	17.5	17.5	17.5
	Δ_*Diff*_	0.4	0.4	0	0	0	0	0.4	0
	*b*	**0**	**0**	**1**	**1**	**1**	**1**	**0**	**1**
	*ξ*_*D*_	HO	HO	F	F	F	F	HO	F
TYR	Δ_*D*_	53.5	53.5	53.9	53.9	53.9	53.9	53.5	53.9
	Δ_*D*_*W*__	53.9	53.9	53.9	53.9	53.9	53.9	53.9	53.9
	Δ_*Diff*_	0.4	0.4	0	0	0	0	0.4	0
	*b*	**0**	**0**	**1**	**1**	**1**	**1**	**0**	**1**
	*ξ*_*D*_	UJ	UJ	QI	QI	QI	QI	UJ	QI
VAL	Δ_*D*_	56.5	56.5	56.9	56.9	56.9	56.9	56.5	56.9
	Δ_*D*_*W*__	56.9	56.9	56.9	56.9	56.9	56.9	56.9	56.9
	Δ_*Diff*_	0.4	0.4	0	0	0	0	0.4	0
	*b*	**0**	**0**	**1**	**1**	**1**	**1**	**0**	**1**
	*ξ*_*D*_	YG	YG	DD	DD	DD	DD	YG	DD

#### 5.1.4 Effect of GenInfoGuard on Statistical and Predictions Measures

The protein secondary structure of ATP8a1 gene was predicted with equal ratio of *α* helix and *β* strand before and after watermarking the protein data set. To build an activity pattern in an amino acid, the secondary structure prediction of an amino acid was represented through the transformation of information from amino acids into hidden units. The whole prediction phenomenon works by exploiting a neural network model that basically imitates the pattern matching capabilities of human brain. The input in this model is amino acids and output is the secondary structure. The performance of GenInfoGuard was measured upon the success probability of prediction of secondary structures before and after applying GenInfoGuard. The success probability (denoted by ℘) is the probability of successfully predicting all types of protein secondary structure. Mathematically,

$$\begin{array}{c} ℘=\frac{P_{\alpha }+P_{\beta }}{N} \end{array}$$ (13)

where *N* is the total number of predicted elements, *P*_*α*_ is the probability of correct prediction of *α* secondary structure, and *P*_*β*_ is the probability of predicting *β* secondary structure. A statistical measure, correlation coefficient Ç, [33] was used to measure the prediction dependance of a variable over other variables. Here Ç_*α*_ is defined for *α* helix and Ç_*β*_ is defined for *β* strand in Equation 14 and Equation 15.

$$\begin{array}{c} {Ç}_{\alpha }=\frac{\left(TP.TN)-(FN.FP\right)}{\sqrt{\left(TN+FN)(TN+FP)(TP+FP)(TP+FN\right)}} \end{array}$$ (14)

$$\begin{array}{c} {Ç}_{\beta }=\frac{\left(TP.TN)-(FN.FP\right)}{\sqrt{\left(TN+FN)(TN+FP)(TP+FP)(TP+FN\right)}} \end{array}$$ (15)

In above equations, the terms *TP, TN, FN, FP* are defined as:

- *TP* (True Positive): Number of those cases that are predicted successfully,
- *TN* (True Negative): Number of those cases that are discarded successfully,
- *FP* (False Positive): Number of those cases that are mistakenly predicted, and
- *FN* (False Negative): Number of those cases that are discarded incorrectly.

All these cases were considered for the calculation of correlation coefficient for both *α* helix and *β* strand prediction. The performance of proposed technique was evaluated in neural network input windows through success probability ℘ and dependency of correlation coefficient Ç on prediction of the secondary structure before and after applying GenInfoGuard. The results of such experiments have been shown in Table 7.

**Table 7:** Performance Measures: Success Probability ℘, and Dependency of Correlation Coefficient Ç on Prediction of the Secondary Structure before and after Applying GenInfoGuard.

**Window Size**	**℘_*O*_**	**℘_*W*_**	**Ç_*α*_(*O*)**	**Ç_*α*_(*W*)**	**Ç_*β*_(*O*)**	**Ç_*β*_(*W*)**
1	0.54	0.54	0.11	0.11	0.14	0.14
3	0.58	0.58	0.22	0.22	0.20	0.20
5	0.61	0.61	0.28	0.28	0.26	0.26
7	0.62	0.62	0.32	0.32	0.28	0.28
9	0.62	0.62	0.33	0.33	0.28	0.28
11	0.62	0.62	0.36	0.36	0.29	0.29
13	0.63	0.63	0.35	0.35	0.29	0.29
15	0.62	0.62	0.35	0.35	0.31	0.31
17	0.62	0.62	0.33	0.33	0.27	0.27

**Fig 2:**
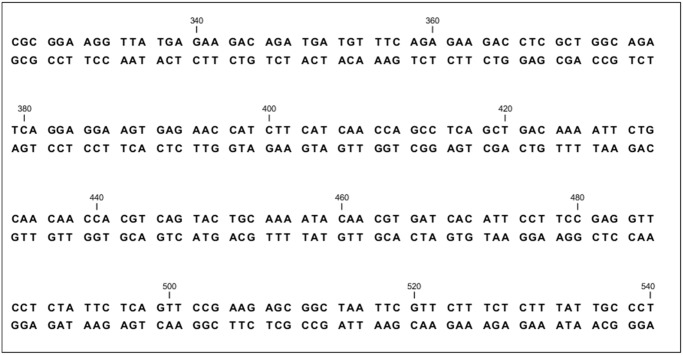
Double Stranded DNA Sequence Alignment before and after Watermarking.

The DNA dataset shown in the Table 4 was experimented with DNA-Crypt v2.0 [3] and GenInfoGuard. If we consider a small DNA sequence CCAGC, it comes out to be the same after marking with GenInfoGuard. While the same DNA sequence results into a different DNA sequence GUUGU after encrypting with DNA-Crypt v2.0. Double stranded DNA sequence alignment was analyzed before and after applying both techniques. DNA-Crypt gives mismatches in such sequence alignment [3]. The results of such experiments have been shown for GenInfoGuard in the Fig. 2. The sequence alignment was performed using CLC bioinformatics tool. Then DNA nucleotide sequence statistics were measured using the same tool for the Fig. 2. The results including count, frequency and change in count Δ(*Count*) and change in frequency Δ(*Frequency*) before and after watermark encoding has been shown in the Table 8. The zero values in the table for Δ(*Count*) and Δ(*Frequency*) show the distortion-free nature of GenInfoGuard.

**Table 8:** Nucleotide Sequence Statistics.

**Nucleotide**	**Count**	**Frequency**	**Δ(*Count*)**	**Δ(*Frequency*)**
Adenine (A)	308	0.262	0	0
Cytosine (C)	310	0.264	0	0
Guanine (G)	293	0.249	0	0
Thymine (T)	264	0.225	0	0
C + G	603	0.513	0	0
A + T	572	0.487	0	0

### 5.2 Robustness Analysis

Robustness analysis of GenInfoGuard is started with a supposition that Alice wants to get ownership protection against the threat model (presented in Section 3). The data recipient, Bob, requires to perform biological processes on genetic data, so he demands high data quality. Bob is willing to receive watermarked data from Alice. Meanwhile, Mallory has malicious intentions and make several attempts to corrupt the data and the watermark encoded in the data.

Robustness of GenInfoGuard was examined through an extensive attack analysis. Our results showed 100% accuracy for the watermark detection. The experiments conducted on DNA dataset have been shown in the Table 4 to demonstrate comparison of DNA-Crypt [6] and GenInfoGuard for data recovery in the best case as well as in the worst case scenarios. Mallory tries to insert, alter and delete 10%, 20%, 30%, 40%, 50%, …, 90% of data. After such attacks, GenInfoGuard recovered 100%, tuples in all the cases. However, DNA-Crypt showed variations in results for insertion, alteration, and deletion attacks on data. GenInfoGuard was also compared with Text Format Based Watermarking (TFBW) [24], Robust and Blind Watermarking (RBW) [23], and Blind and Imperceptible Watermarking (BIW) [34] techniques for watermark detection accuracy with insertion, alteration and deletion attacks. In all these scenarios, GenInfoGuard showed better results.

The computational time of GenInfoGuard is (*l* * *R* * *A*) where *l* is the watermark length, *R* is the total number of tuples in the dataset and *A* is the feature selected for watermarking. Since the number of tuples are very large as compared to features in the databases; therefore, *A* < < *R* and same for watermark length, *l* < < *R*. Therefore, for large databases (*R* termed as *n*), the time complexity of GenInfoGuard for watermark encoding and decoding is *O*(*n*).

Robustness study was conducted empirically for six types of attacks: (1) Insertion attack; (2) deletion attack; (3) alteration attack; (4) sorting attack; (5) additive attack; and (6) counterfeiting attack.

#### 5.2.1 Insertion Attacks

**Fig 3:**
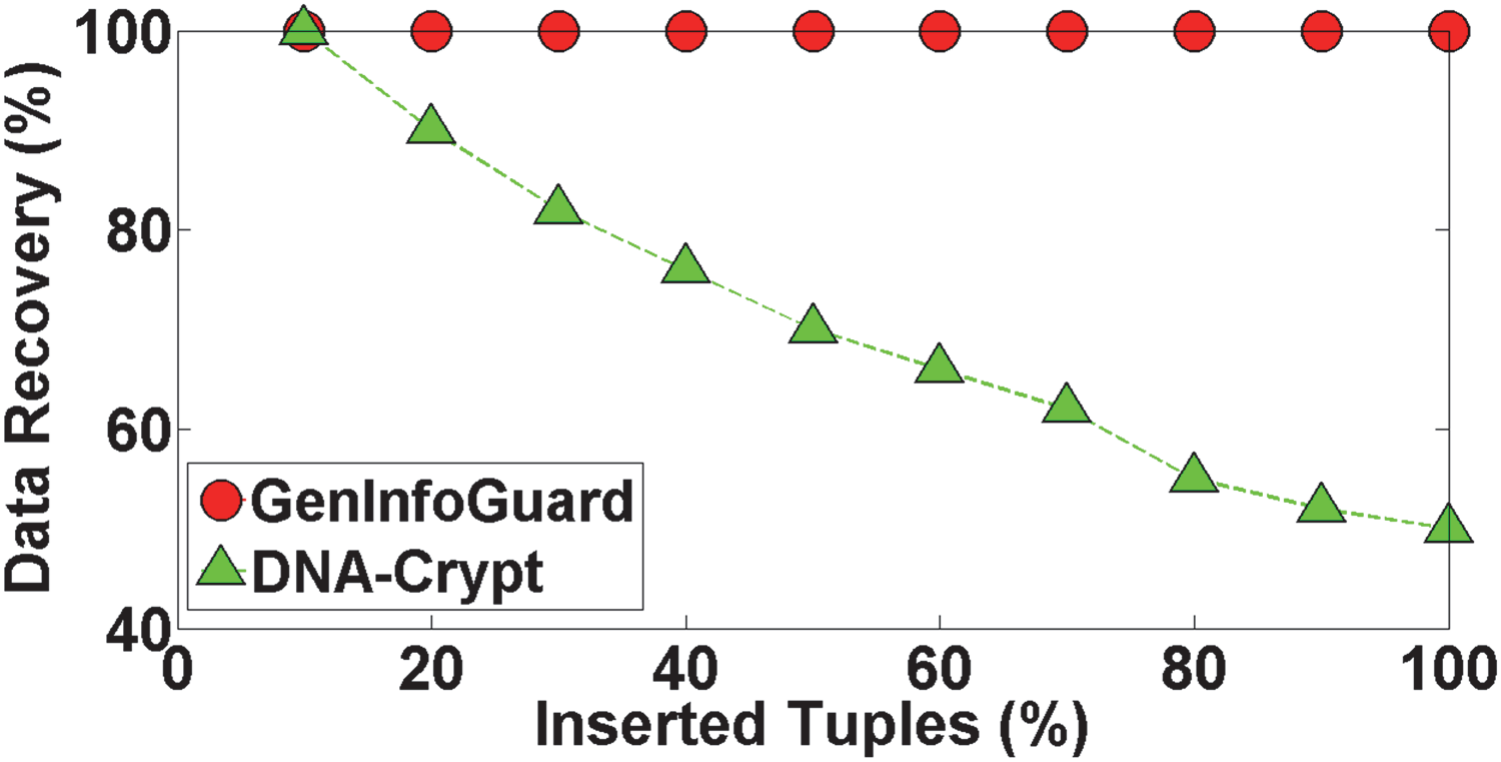
Data Recovery after Insertion Attack.

In this type of attack, Mallory inserts new tuples to corrupt the watermark embedded in the Alice’s data. Insertion of new tuples do not destroy the data integrity and the embedded watermark but may affect the watermark detection rate. GenInfoGuard was observed to be highly resilient against these types of attacks and recovered the watermark and the original data with 100% accuracy even if Mallory inserts 100% new and fake tuples. Data recovery has been shown in the Fig. 3 for GenInfoGuard as well as DNA-Crypt.

**Fig 4:**
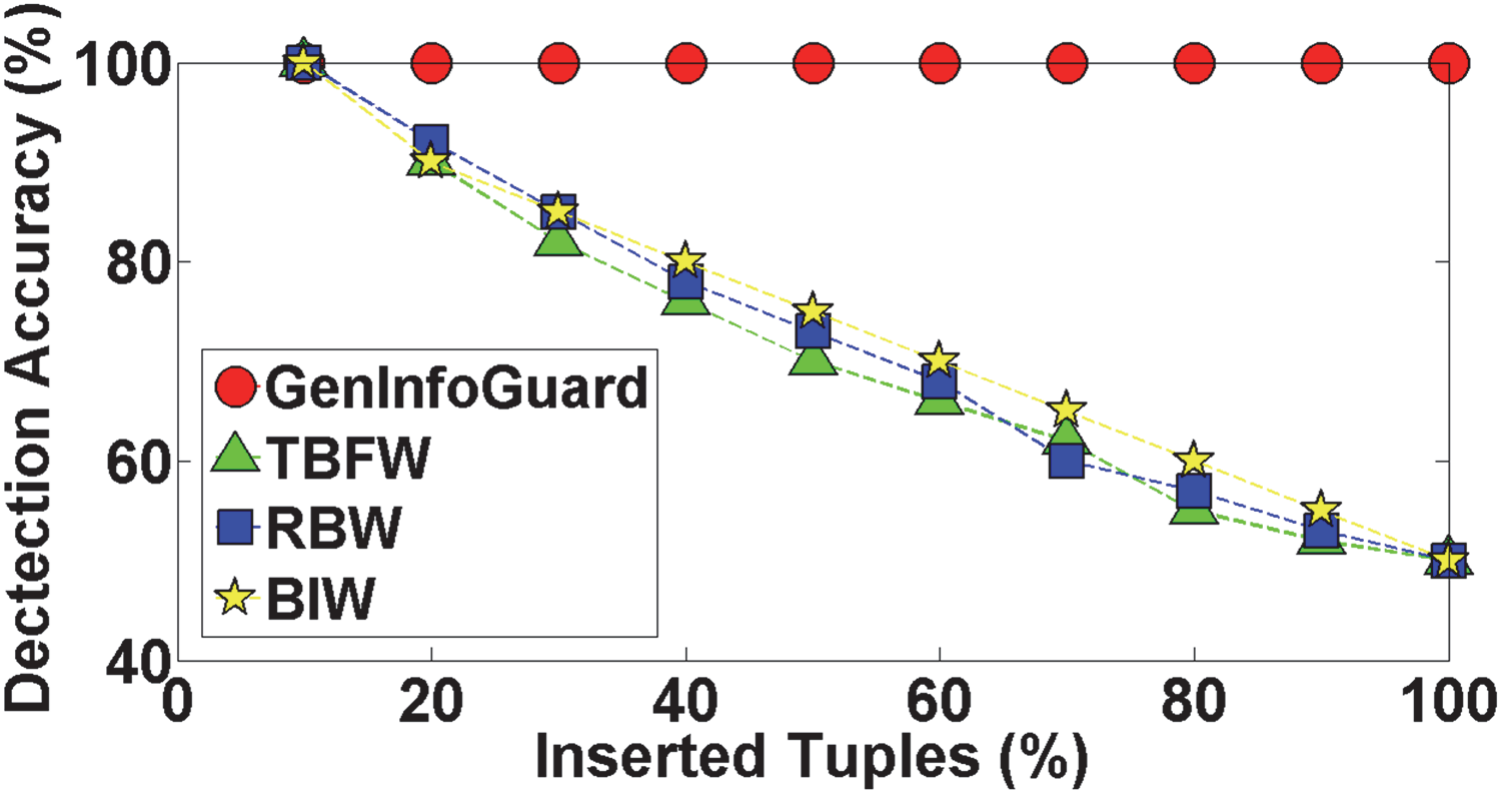
Comparison of Watermark Decoding Accuracy of GenInfoGuard with TFBW, RBW, and BIW after Insertion Attack.

In case of insertion attacks, the success rate of GenInfoGuard was compared with the techniques: TFBW, RBW, and BIW. The watermark decoding success rate was observed to be 100% with GenInfoGuard, between 100% to 50% with TFBW, between 100% to 50% with RBW, and between 100% to 50% with BIW technique as shown in the Fig. 4.

#### 5.2.2 Deletion Attacks

**Fig 5:**
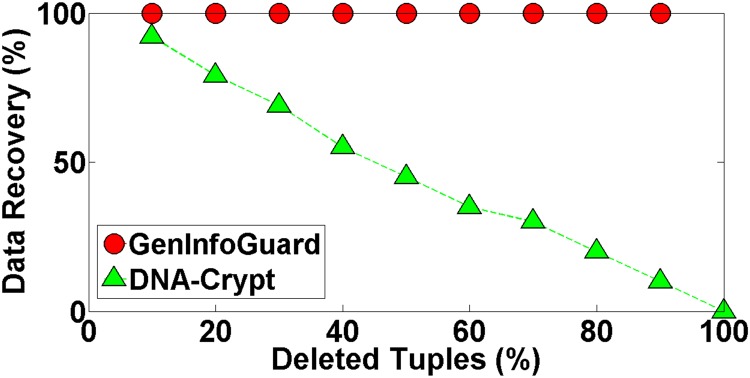
Data Recovery after Deletion Attack.

In such attacks, Mallory deletes a subset of watermarked tuples from the database to corrupt the watermark. The watermark was encoded in the permuted digrams of *n* tuple, so the watermark was extracted even from a permuted digram of a single tuple. Mallory is unable to detect watermark as she is not aware of permuted digrams; therefore, she has the only choice to delete tuples with the concern of preserving the data usefulness of the remaining tuples. Experiments were performed to show the data recovery and the watermark detection. If she can delete *n* − 1 tuples, watermark and the original data would be restored from the remaining 1 tuple of the dataset. In experiments, upto 90% of the data was deleted, so the watermark and the original data retrieved was recovered with 100% accuracy. Data recovery was also observed with 100% success rates under various ranges of deletion attacks as shown in the Fig. 5.

**Fig 6:**
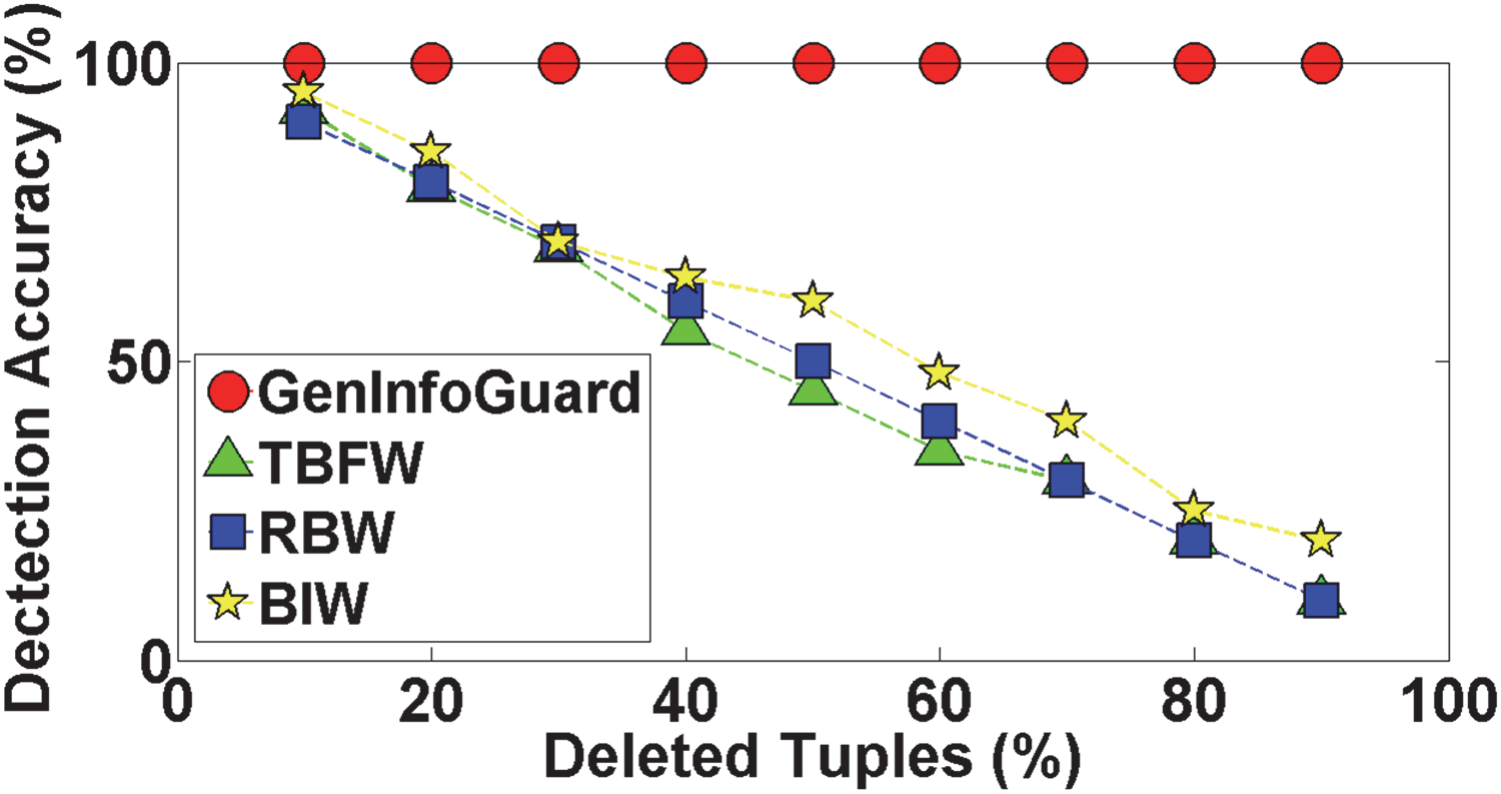
Comparison of Watermark Decoding Accuracy of GenInfoGuard with TFBW, RBW, and BIW for Deletion Attack.

For robustness analysis, GenInfoGuard was also compared with well known irreversible watermarking techniques and success rate of deletion attack with watermark detection was observed. GenInfoGuard showed 100% accurate detection with 90% tuples deletion while TFBW showed between 92% to 0%, RBW between 90% to 0% and BIW between 95% to 0% accuracy with the same number of tuples deletion. GenInfoGuard outperformed other techniques under this type of attack as well. The results of this empirical study has been reported in the Fig. 6.

#### 5.2.3 Alteration Attacks

**Fig 7:**
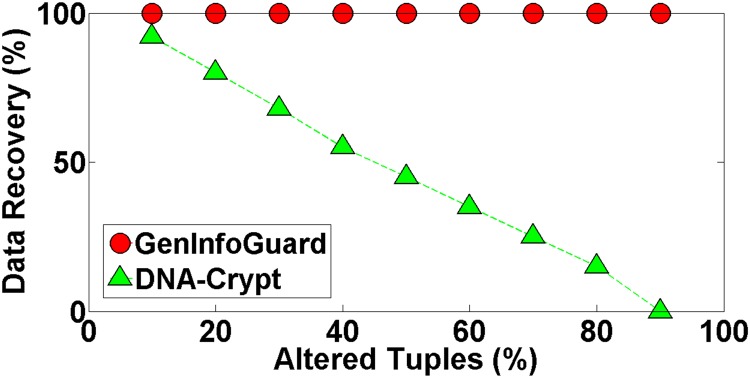
Data Recovery after Alteration Attacks.

**Fig 8:**
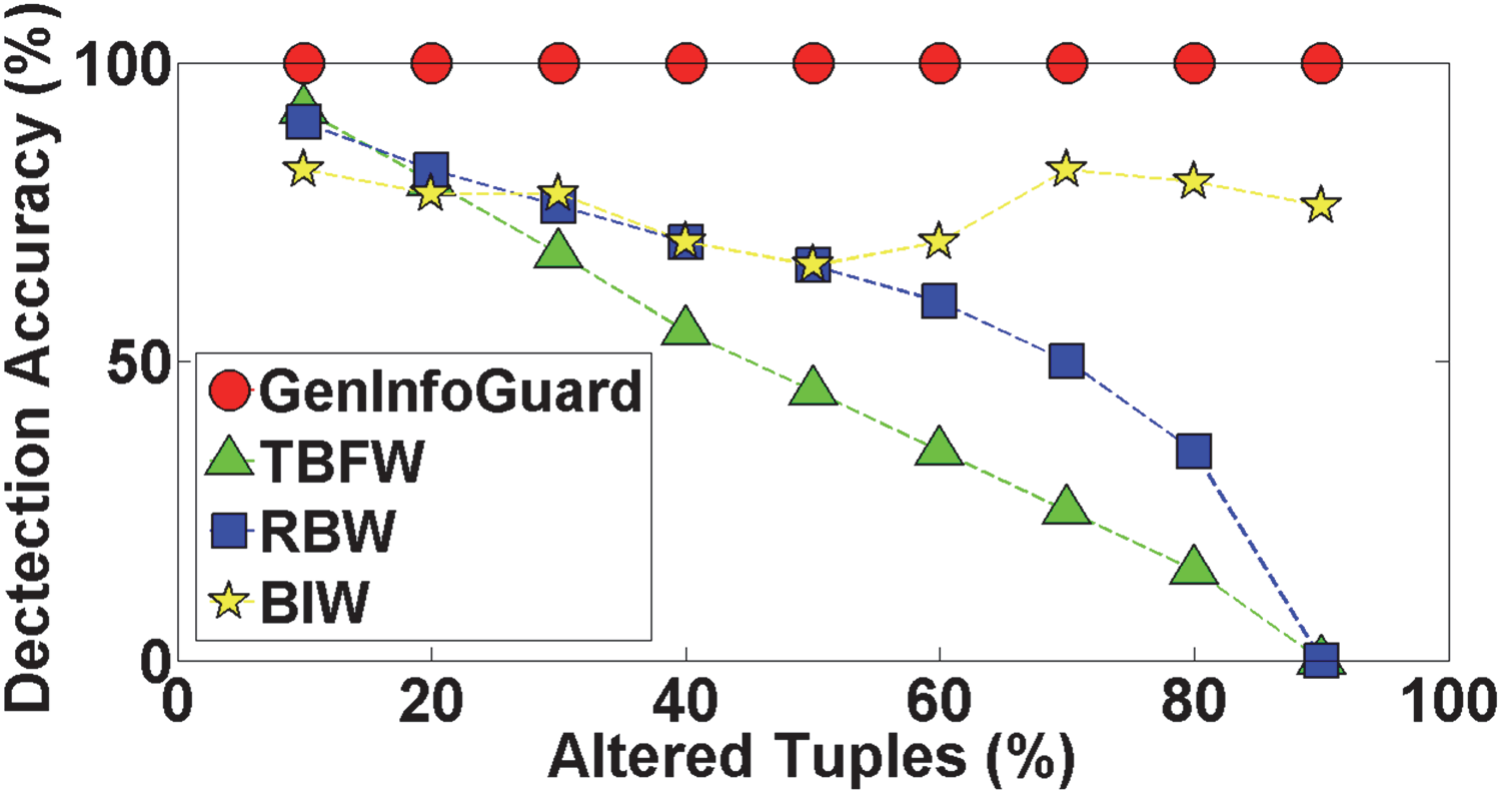
Comparison of Watermark Decoding Accuracy of GenInfoGuard with TFBW, RBW, and BIW for Alteration Attack.

In such attacks, Mallory alters the values of the watermarked data to try to corrupt the watermark. She makes modifications into the data with the intention of destroying the Alice’s watermark. We experimented our technique with such type of attack and observed that the original data was recovered with this type of attack with high success rate. If she can alter *n* − 1 tuples, watermark and the original data would be recovered from the remaining 1 tuple of the dataset. In experiments, GenInfoGuard showed 100% data recovery while DNA-crypt showed less success rate when more than half of the tuples were modified as shown in the Fig. 7. The results of experiments proved that the watermark was detected with accuracy rate of 100%. On the other hand, other techniques did not perform that well as shown in the Fig. 8. The watermark encoding is imperceptible; therefore, Mallory is unable to completely destroy the watermark.

#### 5.2.4 Sorting Attacks

In such type of attack, Mallory can sort the data values in ascending or descending order with the intention of disturbing the encoded watermark. Since in the GenInfoGuard, the watermark was encoded without changing the order of tuples; therefore, it is resilient against sorting attack. GenInfoGuard showed 100% watermark detection with zero probability for success of sorting attacks. In some fragile and zero-watermarking techniques such as [7] and [8], the watermark is embedded in the sorted values of the categorical data; as a result, the watermark detection accuracy is affected due to sorting attacks. The probability of detecting sorting attack has been given in Equation 16.

$$\begin{array}{c} Prob_{sort}=1-\frac{1}{2^{ln(gN)}} \end{array}$$ (16)

#### 5.2.5 Additive Attacks

In additive attacks, Mallory attempts to claim fake ownership of data and embeds a forged watermark into Alice’s data. Mallory’s intentions include: (1) destroying Alice’s watermark; and (2) proving her ownership over the data. However, imperceptible and distortion-free watermark embedding in the permuted digrams makes GenInfoGuard highly robust against additive attacks. As Alice could easily prove her ownership by decoding her own watermark from the data. On the other hand, a certificate can be created as a watermark and registered with a trusted third party, known as certification authority (CA).

In this scenario, Mallory is unable to add her own watermark in the database because it is almost impossible to create the copy of the original certificate that is registered with CA.

Counterfeiting Attacks In this particular attack, Mallory attempts to achieve a forged copy of the Alice’s data so that she can use it in some unauthorized manner. Consider a scenario where Mallory gets access to the watermarked data *D*. However, she is unable to find out the watermark *W* and tries to construct a copy of watermarked data *D* ^′^ with counterfeiting watermark *W*. Consider the encoding function, *Ϝ*_*K*_(*W*, *D*, *P*[Δ_Σ_]), defined below:

$$\begin{array}{c} \begin{array}{cc} & {Ϝ}_{K}\left(W,D,P\left[\Delta_{\Sigma }\right]\right)={Ϝ}_{K}\left(W,D_{1},P\left[\Delta_{\Sigma }\right]\right)∥\\ & {Ϝ}_{K}\left(W,D_{2},P\left[\Delta_{\Sigma }\right]\right)∥\ldots ∥{Ϝ}_{K}\left(W,D_{N},P\left[\Delta_{\Sigma }\right]\right) \end{array} \end{array}$$ (17)

where *K* is the owner defined secret parameter, *P*[Δ_Σ_] is the permuted digrams matrix, and *W* is the watermark. This encoding function encodes watermark in the whole database to minimize the problem of counterfeiting.

Moreover, an imperceptible watermark is encoded in the Alice’s data taking into account a novel distortion-free technique; consequently, the forgery would not be successful and would get detected later with the extraction of Alice’s watermark. Thus, such type of attack has no effect on the Alice’s data.

## Conclusions and Future Directions

Usually, DNA based watermarking techniques for the ownership protection of genetic data introduce modifications into the original data; therefore, information loss occurs. A distortion-free solution is required, that must be robust against benign mutations as well as malicious attacks. To meet this objective, this paper proposed GenInfoGuard: a robust and distortion-free technique for reversible watermarking of genetic data. The benefits of GenInfoGuard are distortion-free and robust solution for ownership protection. The effect on various biological processes after encoding genetic data with GenInfoGuard was analyzed through experiments. The results of these experiments observed before and after the implication of GenInfoGuard confirmed that it did not affect the data quality at all. The robustness of GenInfoGuard was evaluated through attack analysis with different malicious and benign attacks on genetic datasets.
